# Identifying designatable units for intraspecific conservation prioritization: a hierarchical approach applied to the lake whitefish species complex (*Coregonus* spp.)

**DOI:** 10.1111/eva.12247

**Published:** 2015-02-09

**Authors:** Jonathan A Mee, Louis Bernatchez, Jim D Reist, Sean M Rogers, Eric B Taylor

**Affiliations:** 1 Department of Biological Sciences, University of CalgaryCalgary, AB, Canada; 2 Institut de Biologie Intégrative et des Systèmes (IBIS), Université LavalQuébec, QC, Canada; 3 Fisheries and OceansWinnipeg, MB, Canada; 4 Department of Zoology, Biodiversity Research Centre and Beaty Biodiversity Museum, University of British ColumbiaVancouver, BC, Canada

**Keywords:** adaptation, conservation biology, coregonine problem, designatable unit, evolutionarily significant unit, glacial lineages, phylogeography, species complex

## Abstract

The concept of the designatable unit (DU) affords a practical approach to identifying diversity below the species level for conservation prioritization. However, its suitability for defining conservation units in ecologically diverse, geographically widespread and taxonomically challenging species complexes has not been broadly evaluated. The lake whitefish species complex (*Coregonus* spp.) is geographically widespread in the Northern Hemisphere, and it contains a great deal of variability in ecology and evolutionary legacy within and among populations, as well as a great deal of taxonomic ambiguity. Here, we employ a set of hierarchical criteria to identify DUs within the Canadian distribution of the lake whitefish species complex. We identified 36 DUs based on (i) reproductive isolation, (ii) phylogeographic groupings, (iii) local adaptation and (iv) biogeographic regions. The identification of DUs is required for clear discussion regarding the conservation prioritization of lake whitefish populations. We suggest conservation priorities among lake whitefish DUs based on biological consequences of extinction, risk of extinction and distinctiveness. Our results exemplify the need for extensive genetic and biogeographic analyses for any species with broad geographic distributions and the need for detailed evaluation of evolutionary history and adaptive ecological divergence when defining intraspecific conservation units.

## Introduction

The allocation of efforts and resources towards conservation priorities is an important yet challenging aspect of conservation biology (Brooks et al. 2006). Prioritization efforts have largely focused on geographic areas, ecosystems or individual species (Myers et al. 2000). Prioritizing conservation efforts below the species level (e.g. conservation of subspecies, populations or population components) poses particular challenges to managers (O'Brien and Mayr 1991; Allendorf et al. 1997; Taylor et al. 2011; DFO 2013). Nonetheless, diversity below the species level is important because it is the raw material for the generation of new species (e.g. incipient species) (O'Brien and Mayr 1991), it allows the persistence of species following environmental change (i.e. evolution, emigration or extinction) (Davis et al. 2005; Parmesan 2006), and it can involve important local adaptation (e.g. in Pacific salmon populations) (Waples 1991; Fraser and Bernatchez 2001; Hilborn et al. 2003). Several authors have suggested that the appropriate focus for conservation efforts below the species level is the evolutionarily significant unit (ESU) (Ryder 1986; Waples 1991; Waples 1995; Moritz 1994). Defining an ESU in an operational or practical sense rather than in an academic or semantic sense, however, is challenging, and the identification of relevant intraspecific diversity requires a rigorous and repeatable approach (Waples and Gaggiotti 2006).

As an alternative to the ESU concept, the Committee on the Status of Endangered Wildlife in Canada (COSEWIC) uses the concept of the designatable unit (DU), which allows a pragmatic approach that can be applied in the conservation and management of biodiversity below the species level. The DU concept identifies intraspecific units for conservation in cases when recognizing only the species (or subspecies) *per se* likely does not reflect the extent of evolutionarily significant diversity within that species (or subspecies). The DU concept was developed to deal effectively with ‘distinct populations’ in the context of endangered species assessment based on provisions in the *Species At Risk Act* in Canada, which is analogous to, and derived from, the way in which ESUs were developed to deal effectively with ‘distinct population segments’ in the *Endangered Species Act* in the USA. In Canada, guidelines for recognizing DUs have been developed by COSEWIC (2012). According to these guidelines, a DU should be recognized as a unit of intraspecific diversity when it can be identified as discrete from other such units and when it can be identified as significant, where a ‘significant’ unit is one that is important to the evolutionary legacy of the species as a whole and if lost would likely not be replaced through natural dispersion (COSEWIC 2012). An evaluation of discreteness and significance according to these guidelines still requires some interpretation to apply these concepts to a given taxon, and it may be desirable to employ clear and repeatable criteria to guide an objective interpretation of the guidelines (e.g. Taylor et al. 2013).

The DU approach enables recognition of distinct populations when a strictly taxonomic approach is not possible. Distinct populations may be associated with variation in ecology, local adaptation or phylogeographic history that have not been recognized by systematists in the form of taxonomic designations below the species level (e.g. Taylor et al. 2013). Important variation that is not captured by existing taxonomy is likely to be a common component of DU identification. In such cases, it is important to use an approach that provides a clear heuristic to systematically identify populations or population components that warrant DU status. For example, a taxonomic approach fails to capture all the relevant diversity among new world ciscoes (Pisces: Salmonidae, Coregoninae), where there is a lack of correspondence between existing taxonomic designations (e.g. *Coregonus artedi*, *Coregonus hoyi*, *Coregonus kiyi*, *Coregonus nigripinnis*, *Coregonus nipigon* and C*. zenithicus*) and morphological, ecological and genetic diversity (DFO 2013). Caribou (*Rangifer tarandus*) exemplify the potential lack of correspondence between subspecies or ecotype designation and relevant ecological, geographic and genetic diversity (Serrouya et al. 2012; Yannic et al. 2014). In North America, there are five recognized subspecies of caribou (*R. t. dawsoni*, *R. t. groenlandicus*, *R. t. caribou*, *R. t. granti*, and *R. t. pearyi*; Banfield 1961), but demographic and phylogeographic analyses belie many of these taxonomic distinctions (Serrouya et al. 2012; Weckworth et al. 2012). Three ecotypes of woodland caribou (*R. t. caribou*) have been recognized (boreal caribou, shallow-snow mountain caribou, and deep-snow mountain caribou; Heard and Vagt 1998), but the correspondence between ecotypic and genetic variation is even less clear than the correspondence between subspecies and phylogeography (Serrouya et al. 2012). There is clearly a great deal of diversity among caribou populations in North America, but existing taxonomy fails to capture the diversity that is relevant to the evolutionary legacy and potential of the species (COSEWIC 2011).

An additional challenge to identify distinct populations is encountered when a species is geographically widespread and diverse: At what geographic scale do we set boundaries between DUs? Few attempts have been made to apply the DU concept in such challenging cases. For instance, Taylor et al. (2013) studied diverse and broadly distributed populations of lake chub (Pisces: *Couesius plumbeus*). They identified twelve DUs based on phylogenetic lineages, local physiological adaptation to thermal springs and presence in different biogeographic regions (Taylor et al. 2013). In this study, we identify DUs in an even more widespread fish species that occurs across a variable landscape, which displays remarkable variation within and between populations in ecology, life-history evolution and biogeographic history, and which has high taxonomic and phenotypic complexity.

The lake whitefish species complex (*Coregonus* spp.) in Canada provides an excellent opportunity to exemplify the application of the DU approach in a challenging case. This highly studied and data-rich species complex exhibits significant variability in ecology and evolutionary legacy within and among populations distributed across a broad geographic range that defies a strictly taxonomic approach. Coregonines are cold-water fishes common throughout the Holarctic in North America, Europe and Asia (Lindsey and Woods 1970). They support important commercial and recreational fisheries and are the focus of significant worldwide aquaculture operations (Eckmann et al. 1998). Coregonines are a dynamic example of contemporary evolutionary change. Numerous forms (variously described as species, subspecies or ecotypes) have evolved throughout their entire distribution during and after the Pleistocene glaciations (Kirkpatrick and Selander 1979; Bodaly et al. 1991b; Vuorinen et al. 1993; Svärdson 1998; Turgeon et al. 1999; Bernatchez 2004; Ostbye et al. 2006; McDermid et al. 2007). Their broad distribution and successful colonization of lake and river environments following the retreat of glacial ice has contributed to the significant interest of coregonines as a model system for understanding adaptive evolution and ecological speciation (Ostbye et al. 2006; Rogers and Bernatchez 2007; Bernatchez et al. 2010; Vonlanthen et al. 2012; Rogers et al. 2013; Siwertsson et al. 2013). However, the broad distribution and dynamic evolutionary histories of many coregonine species have also resulted in inconsistent taxonomy and disagreements in nomenclature, an issue that is commonly referred to as the ‘coregonine problem’ (Svärdson 1957, 1965; Lindsey et al. 1970; Nikolsky and Reshetnikov 1970; Scott and Crossman 1979; Douglas et al. 2005; McPhail 2007), although perhaps better referred to in the plural (i.e. coregonine problems).

**Figure 1:**
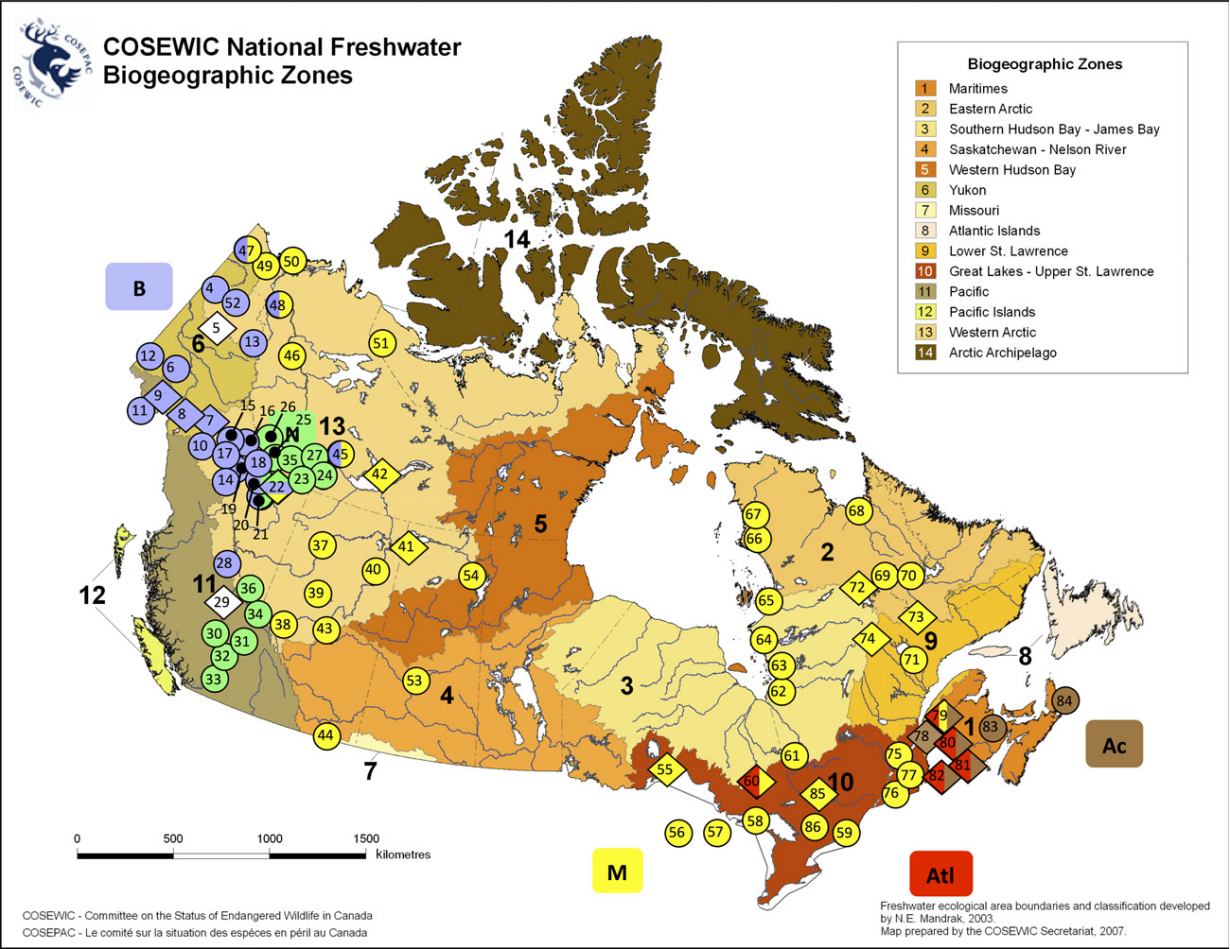
Distribution and phylogeography of lake whitefish in Canada. Locations of lake whitefish populations as indicated in the figure are approximate. Colours of populations correspond to phylogenetic groupings representative of glacial lineages. Blue = Beringian, green = Nahanni, yellow = Mississippi, red = Atlantic, brown = Acadian, white = not available. Locations with species pairs are labelled as diamonds. National Freshwater Aquatic Ecoregions of Canada are colour coded on the map (see legend), which is reproduced from the Committee on the Status of Endangered Wildlife in Canada (COSEWIC) guidelines for recognizing designatable units (COSEWIC 2012). See Table1 for details about the samples and Figure2 for their status as putative DUs.

The lake whitefish is nearly ubiquitous in large freshwater systems in Canada, extending from Yukon Territory to Labrador, inhabiting the lakes and rivers of every province except Prince Edward Island, extending southward into the New England and Great Lake states (USA), and northward onto Victoria Island near Cambridge Bay in the Arctic Archipelago (Fig.1; Scott and Crossman 1979). Lake whitefish is the second most valuable commercial freshwater fish in Canada (Bodaly 1986; DFO 2012). Recognizing lake whitefish DUs has proven difficult when only a few populations are taken into account because the question ‘how different is different’ depends on comparisons with large-scale patterns and magnitudes of diversity (see, for example, Bernard et al. 2009). Hence, a prerequisite for assessment of conservation priorities and management of lake whitefish populations in Canada is an evaluation of the entire lake whitefish species complex across its distribution. Prioritization schemes that take into account evolutionary and phylogenetic distinctiveness (Taylor et al. 2011; Diniz-Filho et al. 2013; Rosauer and Mooers 2013; Jetz et al. 2014; Vokmann et al. 2014) can then be applied to inform management for conservation purposes.

We integrate the broad range of genetic, biogeographic, morphological, physiological and ecological information that is uniquely available for lake whitefish to identify DUs across the Canadian distribution of the lake whitefish species complex. Our study identifies DUs at a geographic scale that is likely to be greater than most DU evaluations (Canada is, geographically, the second largest country on the planet) and, therefore, represents an important example showing that distinct populations can be identified across most geographic scales. Our approach was to interpret existing Canadian guidelines for recognizing DUs using a clear set of hierarchical criteria and to apply these criteria to the lake whitefish species complex. We expect that, as this approach is employed with additional taxa, one of two outcomes will result: (i) it will be validated as a general approach or (ii) it will fail in particular cases, which will precipitate modification(s) to the guidelines and/or the criteria that improve generality.

## Methods

There has been an ongoing refinement of the COSEWIC guidelines for recognizing diversity below the species level, continuing to the present day, and culminating recently in guidelines for recognizing DUs that hinge on identifying *discrete* and *significant* intraspecific units (COSEWIC 2012). The COSEWIC guidelines still require some subjective interpretation, and it is important to be clear regarding how the guidelines are interpreted in any particular situation. Hence, we developed a set of four criteria to interpret and apply the COSEWIC (2012) guidelines to the lake whitefish species complex. Our four criteria distil the COSEWIC guidelines into a clear series of evidence-based decisions, which we used to assess the DU status of lake whitefish populations. We assigned DU status to a population, a group of populations, or a population component if at least one of these criteria applied: (i) the DU comprises a recognized taxonomic entity (i.e. species or subspecies) or is reproductively isolated in sympatry from other DUs (and, hence, qualifies as a biological species), (ii) the DU comprises a genetic lineage with a phylogeographic history that is different from that of other DUs, (iii) the DU has a trait (or suite of traits) resulting from an independent case of local adaptation, and (iv) the DU inhabits a different biogeographic region relative to other DUs. These criteria were intended to capture evolutionarily and ecologically relevant processes at multiple time scales and, in one form or another, were based upon pre-existing philosophies or criteria used to identify or prioritize conservation units below the species level (e.g. Utter 1981; Waples 1995; Allendorf et al. 1997; Moritz 2002).

To make transparent and repeatable decisions about DU status, we must clarify some key points, such as how the terms *discrete* and *significant* from the COSEWIC guidelines (COSEWIC 2012) apply to each criterion. Criterion 1 identifies subspecies as DUs because some kind of discreteness (typically assessed using morphology) is inherent in the recognition of subspecies. Subspecies are, by the strictest definition, a significant part of the evolutionary legacy of a species and are geographically disjunct. Alternatively, populations that are sympatric but reproductively isolated from one another are discrete and evolutionarily significant because such populations are distinct biological species even if they are not recognized subspecies. Hence, DUs were considered discrete and significant if they were found in reproductive sympatry and there was evidence for reproductive isolation between them, even if they have the same taxonomic designation. Reproductive isolation need not be complete to be relevant for diversification (Rogers et al. 2013), and, therefore, we considered evidence for any amount of reproductive isolation in sympatry to be justification for DU status.

For criterion 2, the discreteness of phylogeographic lineages was recognized based on evidence for diagnostic alleles or haplotypes, and the significance of these lineages depended on some measure of statistical support for their discreteness (e.g. in a phylogenetic analysis) and evidence that the origin of such lineages was associated with different refugia that were occupied during the Pleistocene glaciations (e.g. Bernatchez and Wilson 1998; Taylor et al. 2013). Criterion 2 refers only to genetic lineages, allowing for the possibility that a given population (e.g. all the lake whitefish in a single lake) could comprise multiple DUs if that population contained a mix of individuals whose ancestors had different phylogeographic histories.

For criterion 3, our appraisal of local adaptation was dependent on evidence that discreteness among potential DUs in a putatively adaptive trait is genetically controlled and influenced by selection in a particular environment. We equated local adaptation with significant variation as per COSEWIC (2012), given that adaptive traits are likely essential for the persistence of populations within their local environments. If multiple populations were all adapted to a similar (or even identical) environment, each one of these locally adapted populations with an independent origin (or each group of populations with a shared independent origin) was equated with a different DU. The recognition of discreteness among multiple locally adapted populations required only that local adaptation had an independent evolutionary origin. Hence, we avoid any discussion of ‘how different is different’ for populations with unique adaptations to be considered nonexchangeable (sensu Crandall et al. 2000), and focus only on the more tractable question of whether locally adapted traits are independently derived.

For criterion 4, we used *a priori* identification of National Freshwater Biogeographic Zones (NFBZ) based on similarities of fish species communities across watershed boundaries (COSEWIC 2012) to designate discrete groupings. Residence in different NFBZ amounts to significant discreteness between DUs given that such geographic isolation represents distinct biogeographic histories associated with biogeographic provinces with different ecological properties (e.g. McPhail and Lindsey 1970; Mandrak and Crossman 1992).

These criteria were used in a hierarchical (or nested) fashion. The order of the hierarchy was based on the temporal continuum of the evolutionary divergence process: long time-scale evolutionary processes, such as speciation events and the formation of phylogenetic lineages, are reflected in criteria 1 and 2, while shorter time-scale and contemporary ecological and evolutionary processes, such as local adaptation and biogeographic separation, are reflected in criteria 3 and 4. For example, if the application of criterion 1 resulted in the recognition of discrete and significant groups (e.g. there are two recognized subspecies), each of those groups could then evaluated separately based on criterion 2, 3 or 4 to assess whether or not additional discrete and significant groups (likely with a more recent origin) may exist. The hierarchy of our criteria is not intended to atomize a species into the smallest possible units. Instead, the hierarchy is intended as an organizational tool allowing clear identification of DUs based on four key generalizable concepts: reproductive isolation (criterion 1), phylogeographic history (criterion 2), local adaptation (criterion 3) and significant biogeographic separation (criterion 4).

To apply these criteria to the lake whitefish complex in Canada, an extensive lake whitefish literature was reviewed for relevant information about the species with respect to genetics, ecology, morphology, life history, distribution and presence in different aquatic ecoregions (Table1). This is obviously not a complete list of Canadian lake whitefish populations, but we contend that these populations amount to a representative sample of lake whitefish diversity across their distribution. When lakes were sampled multiple times, data from all studies were integrated towards assessing DU status (e.g. multiple DNA markers in phylogenetic studies).

**Table 1:** Lake whitefish populations sampled in Canada and relevant regions of the United States of America. ID: location identification for Figure1. Site: river or lake with province/state abbreviation. *Coregonus lavaretus*: presence of a *C. lavaretus* haplotype and occurrence in limnetic (lim) or benthic (ben) forms (? = uncertain or circumstantial evidence). SP: presence of a confirmed or suspected limnetic–benthic species pair. PG: major phylogeographic groupings (B = Beringian, N = Nahanni, M = Mississippian, At = Atlantic, Ac = Acadian, x = evidence for hybridization between glacial lineages, ? = uncertain inference). BZ, National Freshwater Biogeographic Zones (NFBZs) (see Figure1). DU: Designatable Unit identification (see Figure2). Ref: citations for the information presented for each population.

ID	Site		*C. lavaretus*	SP	PG	BZ	DU	Ref.
1	Yukon R.	AK	Yes		B	n/a	Not assessed	Bernatchez and Dodson (1991); Bodaly et al. (1991b)
2	Minnesota L.	AK	Yes		B	n/a	Not assessed	Bernatchez and Dodson (1991); Bodaly et al. (1991b)
3	Chatanika R.	AK	Yes		B	n/a	Not assessed	Bernatchez and Dodson (1991); Bodaly et al. (1991b)
4	Davis L.	YT			B	6	DU24	Foote et al. (1992)
5	Hanson L.	YT		Y	n/a	6	Extinct	Scott and Crossman (1979)
6	Tatchun L.	YT			B	6	DU24	Franzin and Clayton (1977)
7	Squanga L.	YT	Yes (lim)	Y	B	6	DU1, DU2	Franzin and Clayton (1977), Bernatchez and Dodson (1991); Bodaly et al. (1991b); Foote et al. (1992)
-	Teenah L.	YT	?	Y	n/a	6	Data deficient	Bodaly (1979); Bernatchez et al. (1996)
8	Little Teslin L.	YT	Yes (lim)	Y	B	6	DU3, DU4	Bernatchez and Dodson (1991); Bodaly et al. (1991b)
9	Dezadeash L.	YT	Yes (lim + ben)	Y	B	6	DU5, DU6	Foote et al. (1992); Franzin and Clayton (1977)
10	McClintock L.	YT			B	13	DU23	Foote et al. (1992)
11	Aishihik L.	YT			B	6	DU24	Bernatchez and Dodson (1991); Bodaly et al. (1991b); Foote et al. (1992)
12	Kluane L.	YT			B	6	DU24	Foote et al. (1992); Franzin and Clayton (1977)
13	Margaret L.	YT			B	13	DU23	Foote et al. (1992)
14	Dease L.	BC			B	13	DU23	Foote et al. (1992)
15	Finlayson L.	YT			B	13	DU23	Foote et al. (1992)
16	Frances L.	YT			B	13	DU23	Franzin and Clayton (1977); Foote et al. (1992)
17	Simpson L.	YT			B	13	DU23	Foote et al. (1992)
18	Watson L.	YT			B	13	DU23	Franzin and Clayton (1977); Foote et al. (1992)
19	Wheeler L.	BC			B	13	DU23	Foote et al. (1992)
20	Toobally L.	YT			BxN	13	DU23 × DU25	Foote et al. (1992)
21	Crooked L.	BC			BxN	13	DU23 × DU25	Foote et al. (1992)
22	Lower Liard R.	BC	Yes?	Y	BxNxM?	13	Data deficient	Foote et al. (1992); McPhail (2007); McLeod et al. (1979)
23	Fisherman's L.	NT			N	13	DU25	Foote et al. (1992)
24	Bovie L.	NT			N	13	DU25	Foote et al. (1992)
25	Seaplane L.	NT			N	13	DU25	Foote et al. (1992)
26	Divide L.	NT			N	13	DU25	Foote et al. (1992)
27	Little Doctor L.	NT			N	13	DU25	Foote et al. (1992)
28	Crooked R.	BC			BxN	13	DU23 × DU25	Bernatchez and Dodson (1991); Bodaly et al. (1991b); Foote et al. (1992)
29	Quesnel L.	BC		Y	n/a	11	Data deficient	McPhail and Lindsey (1970)
30	Fraser L.	BC			N	11	DU26	Foote et al. (1992)
31	Aleza L.	BC			N	11	DU26	Foote et al. (1992)
32	Lac la Hache	BC			N	11	DU26	Franzin and Clayton (1977); Foote et al. (1992)Foote et al. (1992)
33	Williams L.	BC			N	11	DU26	Franzin and Clayton (1977); Foote et al. (1992)
34	Summit L.	BC			N	11	DU26	Franzin and Clayton (1977); Foote et al. (1992)
35	McLeod L.	NT			N	11	DU26	Franzin and Clayton (1977); Foote et al. (1992)
36	Moberly L.	BC			N	13	DU25	Franzin and Clayton (1977); Foote et al. (1992)
37	Utikuma L.	AB			N	13	DU25	Foote et al. (1992)
38	Talbot L.	AB			N	13	DU25	Franzin and Clayton (1977); Foote et al. (1992)
39	Lesser Slave L.	AB			M	13	DU27	Foote et al. (1992)
40	Athabasca R.	SK			M	13	DU27	Foote et al. (1992)
41	Athabasca L.	AB			M	13	DU27	Foote et al. (1992)
42	Great Slave L.	NT			M	13	DU27	Franzin and Clayton (1977); Bernatchez and Dodson (1991); Foote et al. (1992)
43	Wabamum L.	AB			NxM?	4	Data deficient	Franzin and Clayton (1977); Bernatchez and Dodson (1991); Foote et al. (1992)
44	Waterton L.	AB			M	7	DU33	Foote et al. (1992); Franzin and Clayton (1977)
45	Fort Simpson	NT			BxNxM?	13	Data deficient	Foote et al. (1992)
46	Fort Good Hope	NT			BxNxM?	13	Data deficient	Foote et al. (1992)
47	East Channel	NT			BxNxM?	13	Data deficient	Foote et al. (1992)
48	Arctic Red R.	NT	Yes		B,M	6	DU22, DU28	Bernatchez and Dodson (1991); Bodaly et al. (1991b)
49	Mackenzie Delt.	NT	Yes		BxNxM?	13	Data deficient	Foote et al. (1992)
50	Fort McPherson	NT			M	13	DU27	Bernatchez and Dodson (1991); Bodaly et al. (1991b)
51	Cox L.	NU			BxNxM?	13	Data deficient	Foote et al. (1992)
52	McEvoy L.	YT	Yes		B	13	DU21	Bernatchez and Dodson (1991); Bodaly et al. (1991b)
53	Jack Fish L.	SK.			M	4	DU32	Bernatchez and Dodson (1991); Bodaly et al. (1991b)
54	South Indian L.	MB			M	5	DU30	Bernatchez and Dodson (1991); Bodaly et al. (1991b)
55	Lake Superior	ON		Y	M	10	DU34	Bernatchez and Dodson (1991); Bodaly et al. (1991b)
56	Lake Michigan	MI			M	n/a	Not assessed	Bernatchez and Dodson (1991); Bodaly et al. (1991b)
57	Lake Michigan	MI			M	n/a	Not assessed	Bernatchez and Dodson (1991); Bodaly et al. (1991b)
58	Lake Huron	MI			M	n/a	Not assessed	Bernatchez and Dodson (1991); Bodaly et al. (1991b)
59	Lake Ontario	ON			M	10	DU34	Bernatchez and Dodson (1991); Bodaly et al. (1991b); Bernard et al. (2009)
60	Como Lake	ON		Y	M	10	DU17, DU18	Bernatchez and Dodson (1991); Bodaly et al. (1991b); Vuorinen et al. (1993)
61	Res. Kipawa	QC			M	10	DU34	Bernatchez and Dodson (1991); Bodaly et al. (1991b)
62	Rupert R.	QC			M	3	DU31	Bernatchez and Dodson (1991); Bodaly et al. (1991b)
63	Eastmain R.	QC			M	3	DU31	Bernatchez and Dodson (1991); Bodaly et al. (1991b)
64	La Grande R.	QC			M	3	DU31	Bernatchez and Dodson (1991); Bodaly et al. (1991b)
65	Great Whale R.	QC			M	3	DU31	Bernatchez and Dodson (1991); Bodaly et al. (1991b)
66	Inukjuak R.	QC			M	2	DU29	Bernatchez and Dodson (1991); Bodaly et al. (1991b)
67	Povungnituk R.	QC			M	2	DU29	Bernatchez and Dodson (1991); Bodaly et al. (1991b)
68	Koksoak R.	QC			M	2	DU29	Bernatchez and Dodson (1991); Bodaly et al. (1991b)
69	Squaw L.	QC			M	2	DU29	Bernatchez and Dodson (1991); Bodaly et al. (1991b)
70	Altikamagen L.	QC			M	2	DU29	Bernatchez and Dodson (1991); Bodaly et al. (1991b)
71	Res. Manic. I	QC			M	2	DU29	Bernatchez and Dodson (1991); Bodaly et al. (1991b)
72	Caniapiscau	QC		Y	M	2	DU7, DU8	Bernatchez and Dodson (1990); Pigeon et al. (1997)
73	Res. Manic. V	QC		Y	M	9	DU9, DU10	Bernatchez and Dodson (1990); Pigeon et al. (1997)
74	Outardes II	QC		Y	M	9	DU11, DU12	Bernatchez and Dodson (1990); Pigeon et al. (1997)
75	St. Lawrence R.	QC			M	10	DU34	Bernatchez and Dodson (1991); Bodaly et al. (1991b)
76	L. Champlain	QC			M	9	DU35	Bernatchez and Dodson (1991); Bodaly et al. (1991b)
77	L. St-Francois	QC			M	9	DU35	Bernatchez and Dodson (1991); Bodaly et al. (1991b)
78	East L.	QC		Y	Ac	9	DU19, DU20	Pigeon et al. (1997); Lu and Bernatchez (1999a,b); Lu et al. (2001)
79	L. Témiscouata	QC		Y	M,At,Ac	1	DU15, DU16	Bernatchez and Dodson (1990); Bernatchez and Dodson (1991); Bodaly et al. (1991b); Pigeon et al. (1997); Lu and Bernatchez (1999a,b); Rogers et al. (2001)
80	Spider L.	ME			At,Ac	n/a	Not assessed	Bernatchez and Dodson (1991); Bodaly et al. (1991b)
81	Musquacook L.	ME			At,Ac	n/a	Not assessed	Bernatchez and Dodson (1991); Bodaly et al. (1991b)
82	Cliff L.	ME		Y	At,Ac	n/a	Not assessed	Bernatchez and Dodson (1990); Bernatchez and Dodson (1991); Bodaly et al. (1991b); Pigeon et al. (1997); Lu and Bernatchez (1999a,b); Lu et al. (2001)
83	Grand L.	NB			Ac	1	DU36	Bernatchez and Dodson (1991); Bodaly et al. (1991b)
84	Mira River	NS			Ac	1	DU36	Bernatchez and Dodson (1991); Bodaly et al. (1991b)
85	Opeongo Lake	ON		Y	M	10	DU13, DU14	Kennedy (1943); Bernatchez and Dodson (1991); Bodaly et al. (1991b)
86	Lake Simcoe	ON		?	M	10	DU34	Scott and Crossman (1979); MacCrimmon and Skobe (1970); Bernard et al. (2009)

In certain cases, insufficient or low-quality data limited our ability to apply the criteria. In these cases, we suggest that our DU designation be used as a first approximation. For instance, we point out populations where there is no *direct* evidence to support a decision to list a given population as a DU, but inductive/deductive reasoning based on *indirect* evidence suggests that the population should be granted DU status.

## Results

**Figure 2:**
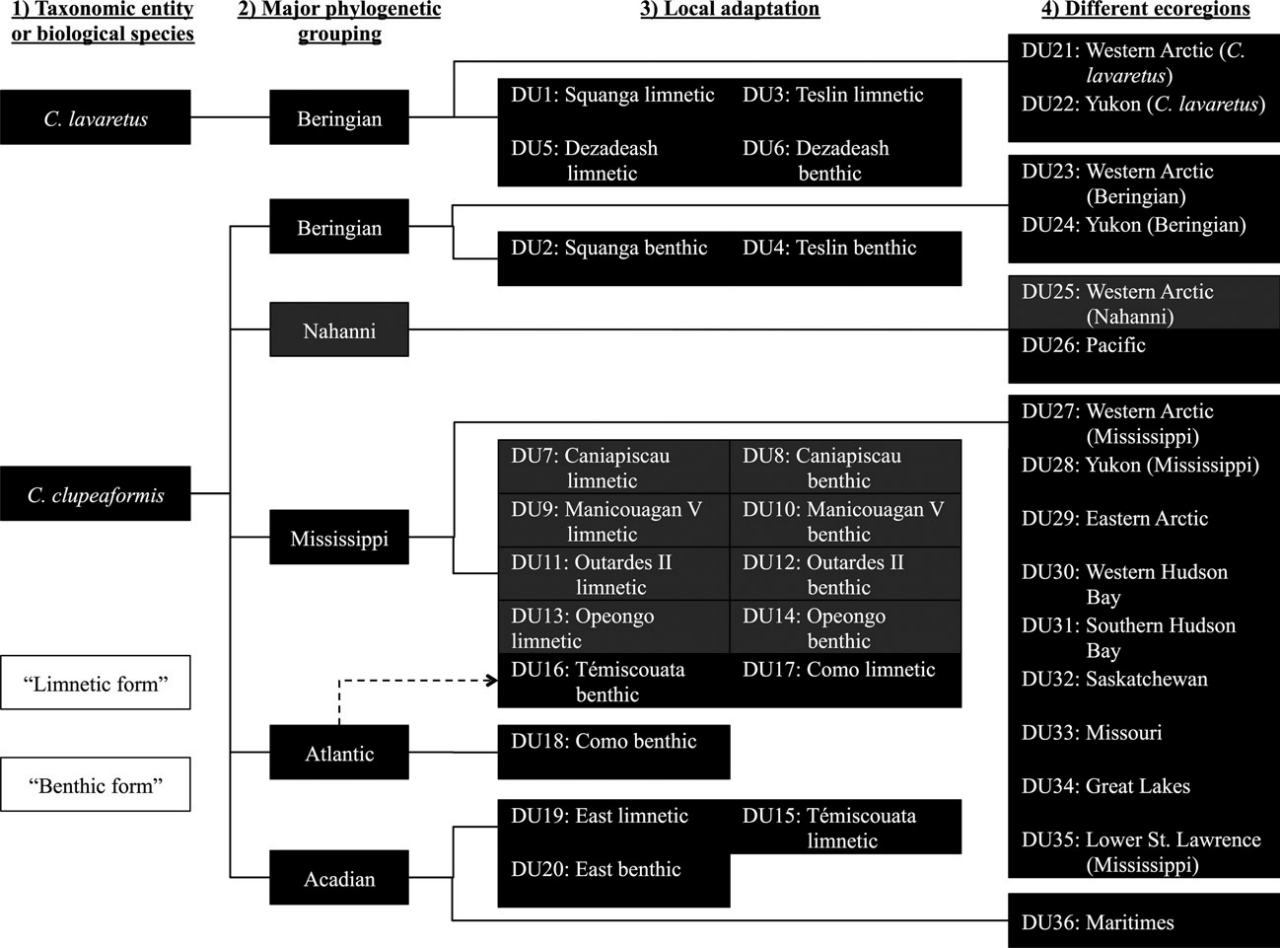
Lake whitefish species complex DU decision chart. The headings at the top, from left to right, reflect the criteria used to identify designatable units (DUs). The boxes show DUs (including data-limited DUs, shown in grey boxes) identified for each of these steps. Lines connecting DUs reflect the hierarchy of steps in the decision process (as opposed on phylogenetic relationships). The characteristic limnetic and benthic forms of lake whitefish are, in general, recognized as biological species, but DU status of these species pairs is actually assigned based on criterion three to reflect the involvement of local adaptation in the evolution of the species pairs.

We identified DUs for Canadian lake whitefish based on available information for 87 populations across its entire distribution range (Table1). Below, we provide the details of the arguments supporting the decision to grant DU status to particular whitefish populations or groups of populations based on each of our four criteria (Fig.2).

### The DU comprises a recognized taxonomic entity or is reproductively isolated from other putative DUs

Allozyme and mtDNA phylogenetic analyses have demonstrated that some North American lake whitefish populations inhabiting northwestern Canada (and Alaska) are phylogenetically more closely related to European Whitefish (*Coregonus lavaretus* Linnaeus) than to North American populations of *C. clupeaformis* Mitchill (Bernatchez et al. 1991; Bodaly et al. 1991b; Bodaly et al. 1994; Bernatchez and Dodson 1994; Sajdak and Phillips 1997). The distribution of *C. lavaretus* extends westward from Siberia into Fennoscandinavia and central alpine European lakes (Walters 1955; Bodaly et al. 1994; Svärdson 1998; Politov et al. 1999, 2000). Populations of *C. lavaretus* were able to disperse to northwestern North America (i.e. Alaska) from Siberia via freshwater or brackish environments across the Bering land bridge that existed during Pleistocene glacial maxima (Lindsey and McPhail 1986; Bernatchez and Dodson 1994). *Coregonus lavaretus* and *C. clupeaformis* overlap and are sympatric in only three Canadian locations that have been investigated: the Arctic Red River, Squanga Lake and Little Teslin Lake (Table1). Within these locations and other locations with sympatry (such as in Alaska), there is evidence of reproductive isolation between *C. lavaretus* and *C. clupeaformis* (Lindsey 1963). Hence, within the North American lake whitefish complex, populations of *C. lavaretus* should be recognized as a discrete and significant unit relative to *C. clupeaformis* (Fig.2).

Morphologically discrete populations of lake whitefish have been reported to occur in sympatry in 18 northern temperate lakes in Canada from the Yukon to Labrador (Lindsey 1963; McPhail and Lindsey 1970; Bodaly 1977; Bernatchez and Dodson 1990; Bodaly et al. 1992; Bernatchez et al. 1996). These so-called species pairs typically include a limnetic form (or ecotype) that is slower growing, matures at an earlier age and size, and lives in the limnetic zone of lakes, and a benthic form (or ecotype) that grows faster and to a larger size, matures at a later age, and lives within the benthic zone of lakes. The limnetic form is often referred to as the ‘dwarf’ form, and the benthic form is often called the ‘normal’ form. There is direct genetic evidence for reproductive isolation between benthic and limnetic forms in six of the 18 lakes purported to contain a species pair (Table2). For example, the progeny from limnetic–benthic hybrid backcross families either died during development or hatched at a suboptimal time, suggesting that genetic incompatibilities and extrinsic postzygotic isolation contribute to reproductive barriers (Rogers and Bernatchez 2006), although the different glacial backgrounds used in those crosses may have exaggerated the level of incompatibility. Limnetic–benthic F1 and backcross hybrids also show increased levels of gene misexpression, including for key developmental genes involved in protein folding, mRNA translation, and transposon reactivation, potentially resulting in developmental abnormalities (Renaut et al. 2009; Dion-Côté et al. 2014), which supports the existence of intrinsic postzygotic reproductive isolation between these limnetic and benthic forms.

**Table 2:** Summary of evidence for reproductive isolation between ecotypes of lake whitefish in 16 Canadian lakes where species pairs have been reported.

Lake or river system	Evidence for reproductive isolation between ecotypes	References
Squanga Lake, YT	Direct genetic evidence	Bodaly (1979); Bernatchez et al. (1996)
Little Teslin Lake, YT	Direct genetic evidence	Bodaly (1979); Bernatchez et al. (1996)
Dezadeash Lake, YT	Direct genetic evidence	Bodaly (1979); Bernatchez et al. (1996)
Teenah Lake, YT	Insufficient data	Bodaly (1979)
Hanson Lake, YT	Insufficient data (extirpated)	Scott and Crossman (1979)
Dragon Lake, YT	Insufficient data (extirpated)	Scott and Crossman (1979)
Lower Liard River, BC	Insufficient data	McPhail (2007); McLeod et al. (1979)
Como Lake, ON	Direct genetic evidence	Bodaly et al. (1991a); Vuorinen et al. (1993)
Opeongo Lake, ON	Indirect morphological evidence	Kennedy (1943)
Lake Superior, ON	Insufficient data	Bodaly et al. (1991b)
Lake Simcoe, ON	Insufficient data	Scott and Crossman (1979); MacCrimmon and Skobe (1970)
Rés. Outardes II, QC	Indirect morphological evidence	Fortin and Gendron (1990)
Rés. Manicouagan V, QC	Indirect morphological evidence	Pigeon et al. (1997)
Rés. Caniapiscau, QC	Indirect morphological evidence	Doyon et al. (1998)
East Lake, QC	Direct genetic evidence	Lu et al. (2001); Rogers et al. (2001); Rogers and Bernatchez (2007); Campbell and Bernatchez (2004); Gagnaire et al. (2013); Pavey et al. (2013)
Lac Témiscouata, QC	Direct genetic evidence	Lu and Bernatchez (1999b); Lu et al. (2001); Rogers et al. (2001); Rogers and Bernatchez (2005); Rogers and Bernatchez (2007); Gagnaire et al. (2013); Pavey et al. (2013); Renaut et al. (2009); Dion-Côté et al. (2014)

There is a characteristic suite of phenotypic traits and genetic changes associated with the limnetic and benthic dichotomy that, in the absence of direct genetic evidence for reproductive isolation, provides indirect evidence for the existence of biological species (Fenderson 1964; Chouinard and Bernatchez 1998; Derome et al. 2006; Bernatchez et al. 2010). In addition to the six lakes with direct evidence for reproductive isolation, there is sufficient phenotypic data from four lakes to provide indirect evidence that the benthic and limnetic forms are reproductively isolated (Table2). Three additional lakes and one large river system purported to contain a species pair are too data deficient to make any inferences regarding the existence of biological species (Table2). Two purported species pairs have been extirpated (Hanson Lake and Dragon Lake; Table2), and two do not conform to the known suite of traits characteristic of the benthic and limnetic dichotomy (Great Slave Lake and Lake Athabasca; not included in Table2).

Overall, there is strong evidence that benthic and limnetic lake whitefish behave as biological species. We have not assigned DU status to ‘limnetic Lake Whitefish’ and ‘benthic Lake Whitefish’ *per se* across the whole distribution of these populations because of the evidence for independent evolution of each benthic and limnetic population as a result of local adaptation (see analysis of criterion 3, below), which justifies the independent DU status of particular benthic and limnetic populations. Nonetheless, the likelihood that each of these independently derived benthic and limnetic ecotypes represents a biological species greatly strengthens the argument for DU status for each locally adapted benthic and limnetic population.

### The DU comprises a genetic lineage with a phylogeographic history that is different from that of other DUs

**Figure 3:**
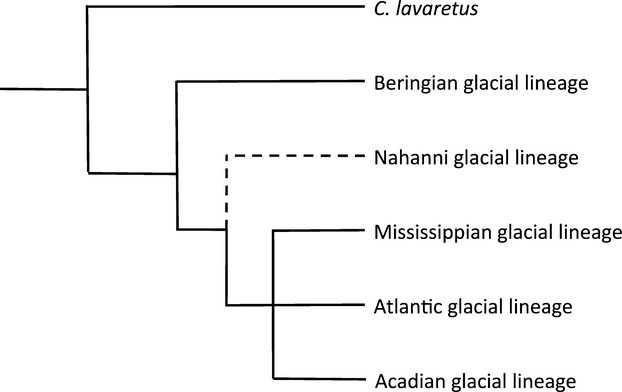
Summary of phylogenetic relationships among lake whitefish glacial lineages. This summary is drawn from the phylogenetic analyses in several studies, many of which use different molecular tools (e.g. allozymes, mitochondrial DNA markers, nuclear markers) to make phylogenetic inferences (Bernatchez and Dodson 1991; Bodaly et al. 1991b; Bodaly et al. 1992; Foote et al. 1992; Bernatchez and Dodson 1994; Bernatchez et al. 1991; Lu et al. 2001). Branch lengths are not to scale. The dashed line represents the data-limited nature of the Nahanni glacial lineage.

Pleistocene glaciations resulted in extreme geographic and temporal isolation of refugial whitefish populations that has driven the evolution of major phylogenetic groupings within *C. clupeaformis* in North America. Lake whitefish could have only survived in a limited number of locations (i.e. glacial refugia) that were not iced over during the repeated advances of glacial ice during the Pleistocene. In North America, there is evidence for four areas that supported refugial lake whitefish populations until the glaciers retreated approximately 15 000–8000 years ago (McPhail and Lindsey 1970; Pielou 1991; Dawson 1992). The Beringian Refugium included most of Alaska and Yukon, as well as a huge area encompassing the beds of two shallow seas – the Bering and the Chukchi seas (McPhail and Lindsey 1970). The Nahanni Refugium consisted of an ice-free corridor that formed a northward pointing peninsula of ice-free land just east of the Rocky Mountains (Prest 1970; Ford 1974). The Mississippi Refugium consisted of ice-free portions of the Mississippi River drainage south of the glacial ice mass (McPhail and Lindsey 1970). The Atlantic Refugium consisted of drainage systems on the Atlantic coast, east and south of the ice sheet, including the coastal plains (which are now submerged), as well as several smaller coastal refugia that persisted on Cape Breton Island, the Gulf of Saint Lawrence, the Gaspé Peninsula, and Newfoundland (Bailey and Smith 1981; Legendre and Legendre 1984; Mandrak and Crossman 1992; Bernatchez 1997). The existence of these glacial lineages of *C. clupeaformis* in North America and their phylogenetic inter-relationships, including with *C. lavaretus* (Fig.3), is based on a diverse range of evidence.

#### Beringian glacial lineage

Lake whitefish populations currently found in Alaska, Yukon, and the Northwest Territories are derived from populations that survived the last glacial maximum in Beringia (Lindsey 1975; Lindsey and McPhail 1986). As described above (criterion 1), *C. lavaretus* likely migrated into the Beringian Refugium from Eurasia during Pleistocene glacial maxima. *Coregonus clupeaformis* also persisted in the Beringian Refugium, and, as a result, lake whitefish in Alaska, Yukon and the Northwest Territories consist of two significantly discrete groupings of mtDNA haplotypes corresponding to *C. lavaretus* and *C. clupeaformis* (Bernatchez and Dodson 1991). The mtDNA sequence divergence of 1.15% between Beringian *C. clupeaformis* and all other North American *C. clupeaformis* is high relative even to the interspecies sequence divergence of 1.8% between *C. nasus* and *C. clupeaformis* (Bernatchez and Dodson 1991, 1994; Crête-Lafrenière et al. 2012). Thus, this relatively deep intraspecific divergence of *C. clupeaformis* populations corresponding to the Beringian glacial lineage suggests that descendants of this lineage represent a discrete and significant group relative to other North American *C. clupeaformis* (Table1, Fig.2).

#### Mississippian glacial lineage

The Mississippi Refugium was the source of most postglacial lake whitefish colonizations (Bernatchez and Dodson 1991). Mississippian lake whitefish overlap with the Beringian glacial lineage in the northwest (Bernatchez et al. 1996; Rempel and Smith 1998; McDermid et al. 2007) and with Atlantic glacial lineages in lakes in southern Quebec and Maine (Pigeon et al. 1997). Thus, postglacial colonization by descendants of this lineage extended over 5 000 000 km^2^. Phylogenetic analyses suggest that the Mississippian glacial lineage diverged from the Beringian lineage approximately 375 000 years ago, and the phylogenetic node separating the Mississippian lineages from Beringian lineages is well supported by both mtDNA bootstrap values (Bernatchez and Dodson 1991, 1994; Lu et al. 2001) and allozyme data (Bodaly et al. 1992). Therefore, descendants of the Mississippian glacial lineage represent a discrete and significant group relative to other North American *C. clupeaformis* (Table1, Fig.2).

#### Nahanni glacial lineage

Foote et al. (1992) examined allozyme data for 43 lake whitefish populations from northwestern North America and found that a discrete genetic cluster inhabited waters in the southwest corner of the Northwest Territories, central British Columbia, and the lakes of the lower Liard River, Tetcela River, Fraser River, upper Peace River and Talbot River. This cluster of populations was hypothesized to contain the descendants of fish that survived the Pleistocene glaciations in the Nahanni Refugium and thus represents a Nahanni glacial lineage of lake whitefish (Bodaly et al. 1992; Foote et al. 1992). The average Nei's genetic distances, based on allozyme data, among populations in the Nahannian glacial lineage and the adjacent Beringian and Mississippian glacial lineages suggest that the Nahannian lineage is more closely related to the Mississippian lineage than to the Beringian lineage (Bodaly et al. 1992). The presence of abrupt allele frequency changes between adjacent populations of different lineages separated by barriers to dispersal (as opposed to clines in allele frequencies over larger distances), and the absence of certain Beringian or Mississippian alleles in any Nahannian populations suggests that neither postglacial selection on specific alleles nor introgression between Beringian and Mississippian lineages is likely explanations for the origin of the Nahannian allozyme signature (Foote et al. 1992). Nonetheless, neither of these alternative explanations can be ruled out with the available allozyme data, and Nei's genetic distance between the Nahannian and neighbouring glacial lineages was low (0.047, Foote et al. 1992). More certainty regarding the significance of a Nahannian glacial lineage requires more detailed genetic data and analysis.

#### Atlantic and Acadian glacial lineages

Populations of lake whitefish in southeastern Quebec and northeastern USA likely originate from Atlantic refugial populations. Evidence from mtDNA, microsatellite and allozyme data identified two lineages of lake whitefish that are distinct from the Mississippian lineage and are referred to as the ‘Atlantic’ and ‘Acadian’ glacial lineages (Bernatchez and Dodson 1991, 1994; Bernatchez et al. 1991; Lu et al. 2001). The Acadian glacial lineage originated from northern refugial populations on the coastal plains of northeastern North America and diverged from the Mississippian glacial lineage approximately 150 000 years ago, while the Atlantic glacial lineage likely originated from southern coastal populations in the Atlantic Refugium and diverged from the Mississippian glacial lineage approximately 18 000–75 000 years ago (Bernatchez and Dodson 1991; Lu et al. 2001; Jacobsen et al. 2012). An analysis of molecular variance (amova) of Mississippian, Atlantic and Acadian populations in eastern Canada found that between 65% and 82% of the total genetic variance among populations could be attributed to genetic differences among the eastern glacial lineages (Lu et al. 2001). Furthermore, mtDNA analysis of populations in eastern regions shows significant phylogenetic divergence of the Atlantic from the Acadian or Mississippian glacial lineages (bootstrap support = 93%: Lu et al. 2001). Descendants of both the Atlantic and Acadian glacial lineages, therefore, represent discrete and significant groups relative to other North American *C. clupeaformis* (Table1, Fig.2).

### The DU has a trait (or suite of traits) resulting from an independent case of local adaptation

Detailed studies of lake whitefish species pairs in several lakes suggest that the limnetic and benthic lake whitefish dichotomy evolved as a result of genetically based physiological adaptations on the part of the limnetic form (e.g. slower growth and earlier maturation) to survive the adverse conditions (e.g. increased predation) in the limnetic environment (Fenderson 1964; Chouinard and Bernatchez 1998; Rogers and Bernatchez 2005; Rogers and Bernatchez 2007; Derome et al. 2006; St-Cyr et al. 2008; Bernatchez et al. 2010; Evans et al. 2012, 2013; Filteau et al. 2013; Hebert et al. 2013; Pavey et al. 2013). Several methods have been applied to test the hypothesis that natural selection is maintaining differences between limnetic and benthic lake whitefish at the adaptive traits known to influence their fitness in the limnetic and benthic trophic niches in nature (Table3). These include tests for departures from neutral expectations in quantitative trait divergence, ‘common garden’ experiments, genome scans and tests for parallel genetic changes in the limnetic ecotype across lake whitefish species pairs. In addition, limnetic lake whitefish are only found in sympatry with the benthic ecotype and only in the absence of other limnetic coregonine fishes such as the cisco (*Coregonus artedi*) (Pigeon et al. 1997), which implicates ecological release in driving divergent natural selection. The accumulated evidence therefore suggests that selection has indeed resulted in the evolution of local adaptation in lake whitefish species pairs.

**Table 3:** Phenotypic differences and evidence for local adaptation in sympatric lake whitefish benthic and limnetic ecotypes (i.e., species pairs) from North American Lakes.

Species pair lakes	Phenotypic difference (limnetic relative to benthic)													Evidence for local adaptation
	Size at maturity (smaller)	Gill raker count (higher)	Lateral line scales	Adipose fin length	Pectoral fin length	Caudal peduncle length	Maxillary width	Caudal peduncle depth	Depth selection (more pelagic)	Burst swimming (more)	Swim direction changes (more)	Age at maturity (younger)	Growth rate (slower)	
Squanga Lake, YT	X	yes	X	X	yes			X	yes					
Little Teslin Lake, YT	yes	yes	X	X	X		X	X	yes			yes	X	
Dezadeash Lake, YT	X	yes	X	yes	X		X	X	yes			X	X	
Como Lake, ON	yes	X	yes	yes	X									
Opeongo Lake, ON	yes	X	yes		X			X	yes			yes	yes	
Rés. Outardes II, QC	yes	X	yes		X	yes	X	yes				yes	yes	
Rés. Manicouagan V, QC	yes													
Rés. Caniapiscau, QC	yes								yes			yes	yes	
East Lake, QC	yes	X	X	X	X	X	X	X	X	X	X	yes	yes	Qst-Fst, Common garden, Genome scan
Lac Témiscouata, QC	yes	yes	X	X	X	X	X	X	yes	yes	yes	yes	yes	Qst-Fst, Common garden, Parallelism
Cliff Lake, ME	yes	yes	yes	X	X	yes	X	yes	X	X	yes	yes	yes	Qst-Fst, Genome scan, Parallelism
Indian Pond, ME	yes	yes	X	X	X	X	X	X	X	X	X	yes	yes	Qst-Fst, Genome scan, Parallelism

A “yes” indicates the presence of the specified morphological, behavioural, or physiological difference in each lake containing a species pair.

An X indicates no significant difference, and a blank indicates lack of information.

Data in this table are from the following sources: Kennedy (1943), Lindsey (1963), Fenderson (1964), Bodaly (1979), Fortin & Gendron (1990), Vuorinen *et al*. (1993), Bernatchez *et al*. (1996), Pigeon *et al*. (1990), Doyon *et al*. (1998), Lu & Bernatchez (1999a), Rogers *et al*. (2002), Bernatchez (2004), Campbell & Bernatchez (2004), Rogers & Bernatchez (2009), Derome *et al*. (2006), Rogers & Bernatchez (2007), Jeukens *et al*. (2009), Filteau *et al*. (2013).

Given the similarity in phenotypes across lakes containing the limnetic–benthic lake whitefish species pairs, it is likely that very similar ecological conditions produced the selective pressures in each lake that led to the limnetic–benthic divergence. There are, however, two lines of evidence suggesting that each species pair represents an independent origin of local adaptation. First, the limnetic and benthic lake whitefish ecotypes are polyphyletic, each species pair having arisen independently, and in some cases from different ancestral glacial lineages or species (Bernatchez et al. 1996; Pigeon et al. 1997). These independently derived limnetic and benthic ecotypes have arisen via multiple modes of divergence – the species pairs in Squanga Lake, Little Teslin Lake, Como Lake and Lac Témiscouata all had the opportunity for some divergence in allopatry while isolated in different glacial refugia, while other species pairs are likely derived from intralacustrine divergence of sympatric ecotypes (Bernatchez et al. 1996; Pigeon et al. 1997). Second, limnetic and benthic ecotypes are similar but variable in phenotype across populations (Table3). This phenotypic variability includes traits that are likely neutral in terms of adaptation to the limnetic or benthic environment (e.g. lateral line scales, adipose fin length), suggesting the existence of neutral divergence between populations of a given ecotype. There is also variability across species pairs in the pattern of phenotypic divergence in adaptive traits (Table3). For example, the benthic–limnetic divergence in Squanga Lake involves divergence in gill raker number, but not in size at maturity, age at maturity or growth rate, while the benthic–limnetic pair in East Lake involves divergence in size at maturity, age at maturity and growth rate, but not in gill raker number (Bernatchez et al. 1996; Pigeon et al. 1997). Furthermore, the amount of divergence in the traits that differentiate the two ecotypes in a given lake is directly related to the specialization and availability of trophic niches in that lake (Landry et al. 2007; Landry and Bernatchez 2010), and there is a negative correlation across lakes between the extent of gene flow and trait divergence between the two ecotypes (Chouinard et al. 1996; Chouinard and Bernatchez 1998; Lu and Bernatchez 1999a; Renaut et al. 2011).

In summary, the species pairs in different lakes have differentiated to different degrees, and this differentiation has taken multiple trajectories resulting from different modes of divergence, but the divergence in all cases is likely the result of local adaptation. Hence, there is a strong argument that each limnetic and benthic ecotype in each lake represents a unique component of the entire diversity of the lake whitefish species complex. As a result, both ecotypes in each species pair for which reproductive isolation has been observed or inferred (see section The DU comprises a recognized taxonomic entity or is reproductively isolated from other putative DUs, above) should be considered discrete and significant units of lake whitefish diversity (Table1, Fig.2).

### The DU inhabits a different biogeographic region relative to other putative DUs

National Freshwater Biogeographic Zones represent different biogeographic regions within Canada based on similarities of fish species communities across watersheds, but constrained by the five primary watersheds of Canada (Mandrak 2003). The lake whitefish species complex exists in 11 of the 14 aquatic NFBZs (Figs1 and 2). *Coregonus lavaretus* exists in two NFBZs (western Arctic, Yukon). Of the *C. clupeaformis* glacial lineages, the Beringian glacial lineage exists in two NFBZs (western Arctic, Yukon), the Nahanni glacial lineage exists in two NFBZs (western Arctic, Pacific), the Mississippian glacial lineage exists in ten NFBZs (western Arctic, Eastern Arctic, Yukon, Saskatchewan, Missouri, Western Hudson Bay, Southern Hudson Bay, Great Lakes, Lower St. Lawrence, Maritimes), the Atlantic glacial lineage exists in three NFBZs (Great Lakes, Maritimes, Lower St. Lawrence) and the Acadian glacial lineage exists in a single NFBZ (Maritimes). *Coregonus lavaretus* and the Mississippian, Beringian, and Nahanni glacial lineages of *C. clupeaformis* coincide in the western Arctic NFBZ, making the western Arctic the most diverse NFBZ in terms of phylogeographic origins. Lake whitefish populations (or groups of populations) occupying different ecoregions within each taxonomic entity and within each glacial lineage deserve DU status (Fig.2), although this designation might be moot if a population is already recognized as a DU according to a prior criterion (e.g. all populations in the Atlantic glacial lineage are involved in local adaptation and species pair divergence).

According to our review of the available literature, Whitefish in Canada (including both *C. clupeaformis* and *C. lavaretus*) comprise 36 DUs (Fig.2). We identified DUs using a stepwise hierarchical approach such that populations, groups of populations or population components identified as ‘discrete and significant’ groups according to criterion 1 were then subdivided using criterion 2, and so on using subsequent criteria. We identified two discrete and significant groups based on recognized taxonomic entities which qualify as biological species (i.e. criterion 1), five discrete and significant groups based on phylogeographic lineages (i.e. criterion 2), twenty discrete and significant groups based on suites of traits that represent local adaptation (i.e. criterion 3), and 16 discrete and significant groups based on National Freshwater Biogeographic Zones (i.e. criterion 4).

## Discussion

Lake whitefish in North America are taxonomically complex, have a widespread geographic distribution, and display high variability within and among populations in phylogeographic history and locally adapted traits. In the present study, we used a set of hierarchical criteria to guide the delineation of 36 DUs that, according to the available data, encompass the evolutionary legacy and evolutionary potential of lake whitefish in Canada. These criteria provided a clear heuristic for distinguishing lake whitefish DUs, and as such, are well suited to the goal of interpreting COSEWIC guidelines for recognizing evolutionarily and ecologically relevant variation in a practical way for conservation assessment.

Our criteria for evaluating DU status are effective because they focus on aspects of intraspecific diversity that are ‘designatable’ from the human perspective, thereby avoiding, as much as possible, difficulties related to grey areas where a threshold of discreteness or difference may be difficult to justify. We contend, however, that the aspects of intraspecific diversity captured by our criteria also represent biological variation important to the species itself because our criteria are based on biologically relevant concepts (i.e. reproductive isolation, phylogeographic history, local adaptation and biogeographic separation). We also contend that, insofar as our criteria provide a clear interpretation of the COSEWIC guidelines for recognizing DUs, our approach is a practical and effective way to provide the basis for conservation prioritization in the real world. Our evaluation of lake whitefish DUs demonstrates that this approach can be applied to a taxon with a very large geographic range, great ecological and phylogeographic diversity, and a complex taxonomy. We have demonstrated that intraspecific complexities, such as the repeated evolution of locally adapted sympatric lake whitefish species pairs, can be accommodated by this approach to identifying DUs.

We note that recognition of DUs based on COSEWIC National Freshwater Biogeographic Zones (i.e. criterion 4) is, in practice, not applicable outside of Canada. The principle of using biogeographic provinces to identify portions of species distributions that are worthy of DU status is, nonetheless, broadly applicable to other species in other jurisdictions. The necessary first step is to identify biogeographic provinces that are relevant to the species of interest across the geographical range of interest. For example, in Canada, COSEWIC recognizes National Freshwater Biogeographic Zones that are intended to be relevant to freshwater fish species, Terrestrial Amphibians and Reptiles Faunal Provinces that are intended to be relevant to amphibians and reptiles, and National Ecological Areas that are intended to be relevant to terrestrial mammals (COSEWIC 2012). In other jurisdictions, the evaluation of freshwater species could be based on the biogeographic provinces identified by Abell et al. (2008) and available at http://www.feow.org.

Our approach to recognizing DUs is generalizable to other species with similarly broad geographic ranges or complex taxonomies, as well as to species with lesser geographic ranges and simpler taxonomies. We emphasize, however, that the four criteria employed in the present study were used because they were relevant to interpreting the COSEWIC guidelines and identifying intraspecific diversity in the context of lake whitefish. In other taxa, it may be appropriate to use only a subset of these criteria or to use additional criteria based on other general concepts that are relevant to identifying intraspecific diversity (e.g. range disjunction). For example, Taylor et al. (2013) used a similar approach (i.e. clear criteria to interpret the COSEWIC guidelines) to recognize twelve DUs among populations of lake chub (*Couesius plumbeus*) based on phylogenetic lineages, local physiological adaptation to thermal springs and presence in a different NFBZ. Lake chub display a great deal of variability in ecology and evolutionary legacy among populations distributed across a broad geographic range that defies a strictly taxonomic approach to recognizing DUs. In sum, the lake whitefish and lake chub cases demonstrate the utility and efficiency of an approach based on specific criteria to identify discrete and evolutionarily significant elements of diversity below the species level. An important caveat is that greater geographic scale, higher diversity and more complexity necessitates much more (and more detailed) phylogeographic, ecological and life-history data. This may not be practical in cases where financial resources are a limiting factor (e.g. assessing a widespread species in developing nations) but should nevertheless represent an ideal outcome to be used for guiding research into the nature and structure of diversity for conservation purposes.

There may be additional diversity among lake whitefish populations that we did not consider in the present study, but that could be important for a future evaluation of DUs. For example, it might be argued that separation of populations into phyletic provinces is a relevant aspect of the diversity of lake whitefish. Community phylogenetic methods (e.g. Morlon et al. 2011) could be used to identify phyletic provinces based on phylogenies of fish taxa across the distribution of lake whitefish. These phyletic provinces could be used in an analogous way to NFBZ's to identify DUs. In addition, there may be differences among populations or population segments that are not of evolutionary importance, but that may be of great importance for fisheries managers. For instance, once DUs are identified, nonevolutionary criteria that account for commercially relevant differences in life-history or population-level traits could be added to our approach to assist managers in finer-scale evaluations and monitoring.

There are a variety of principles and methodologies that could be used to prioritize conservation efforts among the lake whitefish DUs identified herein (e.g. Allendorf et al. 1997; Taylor et al. 2011; Diniz-Filho et al. 2013; Rosauer and Mooers 2013; Jetz et al. 2014; Vokmann et al. 2014). Allendorf et al. (1997) ranked conservation priority based on biological consequences of extinction and risk of extinction. In the case of lake whitefish, evolutionary divergence in limnetic and benthic ecotypes has been determined by the conditions of the lake (Chouinard et al. 1996; Chouinard and Bernatchez 1998; Lu and Bernatchez 1999a; Landry et al. 2007; Landry and Bernatchez 2010), by standing genetic variation of the colonizers (Rogers and Bernatchez 2005; Barrett and Schluter 2008), and by historically contingent factors that promote their differentiation (Lu et al. 2001; Rogers et al. 2001; Rogers and Bernatchez 2006). The limnetic ecotype and the conditions that led to its divergence therefore differ from one environment to the next. Given the data (see Table3), it would likely be unwise to assume that if one limnetic population was lost it could be created anew (Bernatchez 1995). For example, in analogous species pairs such as in threespine stickleback (*Gasterosteus aculeatus*), alterations of natural habitat, such as the introduction of an invasive species, resulted in the so-called speciation in reverse or collapse of a species pair (Taylor et al. 2006). There is little apparent prospect of recovery for collapsed species pairs (Gilman and Behm 2011). Furthermore, the introduction of ciscoes is a particularly important threat to the persistence of limnetic lake whitefish populations, and COSEWIC has listed the limnetic ecotype in Squanga Lake, Little Teslin Lake and Dezadeash Lake as populations of ‘special concern’ due largely to this threat (COSEWIC 1987). Hence, the likelihood of non-native species introductions may present the greatest risk of extinction for lake whitefish species pairs.

Evolutionary and phylogenetic distinctiveness are fundamentally important aspects of biodiversity (Diniz-Filho et al. 2013), and several prioritization schemes rank conservation priority based on distinctiveness (Taylor et al. 2011; Rosauer and Mooers 2013; Jetz et al. 2014; Vokmann et al. 2014). Here, the few *C. lavaretus* populations in Canada clearly rank high in terms of genetic distinctiveness and therefore ought to also rank high in terms of conservation priority. Among *C. clupeaformis* DUs, the Beringian lineage is the most divergent among the glacial lineages and therefore deserves the highest conservation priority.

Our application of the DU concept to lake whitefish species complex in Canada exemplifies the need for extensive genetic and phylogeographic analyses for species with broad geographic distributions, and the need for detailed evaluation of adaptive ecological divergence when defining intraspecific conservation units in cases where a strictly taxonomic approach will not account for the full range of evolutionarily significant diversity. The identification of DUs based on local adaptation is a prerequisite for ecosystem-based management, wherein managers are able to identify both specific taxa and specific environments that are important for the conservation of within-species diversity. We recommend this approach for species with a high degree of intraspecific diversity, especially where there is a lack of correspondence between taxonomy and ecological, morphological, ecological or genetic diversity. For example, twelve DUs have been proposed across the widespread distribution of caribou in Canada (COSEWIC 2011), but there are important gaps in the available genetic, morphological, ecological and behavioural data that prevent clear phylogeographic inferences or robust assessments of local adaptation. Pending the availability of such data, the application of hierarchical criteria associated with (i) phylogenetic lineages, (ii) range disjunction, (iii) local adaptation and (iv) biogeographic regions would likely provide a clear argument for DU designations among caribou populations in Canada where the current taxonomic characterization is proving inadequate (Banfield 1961; Cronin 1992; Heard and Vagt 1998; Cronin et al. 2005; McDevitt et al. 2009; COSEWIC 2011; Festa-Bianchet et al. 2011; Klutsch et al. 2012; Serrouya et al. 2012; Weckworth et al. 2012). New world ciscoes, for which there is a great deal of data on morphological, ecological and genetic diversity among populations (DFO 2013), are also an excellent candidate for the application of this approach. Finally, this hierarchical framework could also inspire the definition of conservation units for any species with complex phenotypic and genetic diversity from any country.
